# Does Interdisciplinary Research Lead to Higher Citation Impact? The Different Effect of Proximal and Distal Interdisciplinarity

**DOI:** 10.1371/journal.pone.0135095

**Published:** 2015-08-12

**Authors:** Alfredo Yegros-Yegros, Ismael Rafols, Pablo D’Este

**Affiliations:** 1 Centre for Science and Technology Studies (CWTS), Leiden University, Leiden, The Netherlands; 2 *Ingenio* (CSIC-UPV), Universitat Politècnica de València, València, Spain; 3 SPRU—Science and Technology Policy Research, University of Sussex, Brighton, England; 4 Observatoire des Sciences et des Téchniques (OST-HCERES), Paris, France; Katholieke Universiteit Leuven, BELGIUM

## Abstract

This article analyses the effect of degree of interdisciplinarity on the citation impact of individual publications for four different scientific fields. We operationalise interdisciplinarity as disciplinary diversity in the references of a publication, and rather than treating interdisciplinarity as a monodimensional property, we investigate the separate effect of different aspects of diversity on citation impact: i.e. *variety*, *balance* and *disparity*. We use a Tobit regression model to examine the effect of these properties of interdisciplinarity on citation impact, controlling for a range of variables associated with the characteristics of publications. We find that variety has a positive effect on impact, whereas balance and disparity have a negative effect. Our results further qualify the separate effect of these three aspects of diversity by pointing out that all three dimensions of interdisciplinarity display a curvilinear (inverted U-shape) relationship with citation impact. These findings can be interpreted in two different ways. On the one hand, they are consistent with the view that, while combining multiple fields has a positive effect in knowledge creation, successful research is better achieved through research efforts that draw on a relatively proximal range of fields, as distal interdisciplinary research might be too risky and more likely to fail. On the other hand, these results may be interpreted as suggesting that scientific audiences are reluctant to cite heterodox papers that mix highly disparate bodies of knowledge—thus giving less credit to publications that are too groundbreaking or challenging.

## Introduction

The last decades have seen a surge of interdisciplinarity in science policy discourse, as well as an increase in the explicit promotion of interdisciplinary research (IDR) virtually across all scientific fields [1–3]. Promotion policies have included programmes specifically funding ‘interdisciplinarity’ via match-making events such as the National Academies Keck Futures Initiative (NAKFI, http://www.keckfutures.org [4]) or via graduate programmes such as the Integrative Graduate Education and Research Traineeship (IGERT, www.igert.org [5]). More widely, interdisciplinarity has been seen as a highly positive criterion for the most prestigious, high-risk/high-reward grants. A prominent example of the latter are the grants of the new European Research Council (ERC), which ‘aim to support 'Frontier Research', i.e. ‘proposals of an interdisciplinary nature which cross the boundaries between different fields of research’, ‘addressing new and emerging fields’ or ‘introducing unconventional, innovative approaches and scientific inventions’ [6] (p.12).

The assumption underlying these policies is that IDR brings forth more scientific breakthroughs, fosters innovation and helps address societal problems. However, there is little systematic evidence showing that IDR is ‘better’ on its own sake and hence should be specifically funded or promoted by policies that counter or reduce the ‘disciplining’ pressures of disciplines. We would argue that IDR is also viewed positively because it is congruent with the *zeitgeist* of our time, what Zygmunt Bauman calls *liquid modernity* [7], which according to Hoffmann embraces hybridization, deterritorialization, nomadism, diasporism or outsiderness [8].

There are indeed many narratives of successful research, and particularly, major breakthroughs that resulted from IDR (e.g. see Hollingsworth [9], on discoveries in the Rockefeller Center). But there are also plenty of cases of unsuccessful IDR (possibly less reported), such as the Human Biology Program in the mid-1920s or the Human Ecology Program at the beginning of the 1940s also within the Rockefeller Foundation [10] or some other failed IDR projects in medical areas [11]. Hence, it is argued that one should not jump to the conclusion that overall science would "improve" if research were more interdisciplinary. Evidence on whether IDR is more or less "successful" is scarce, messy and inconclusive. Equally, the same lack of inconclusive evidence is found about the benefits of diversity at every level (nation, city, groups, etc.) [12]. This has led a number of scholars such as Jacobs and Frickel [1] (p. 44) to take a sceptical stance on the ‘superiority’ of IDR as a form of research:

*‘The widespread attention that administrators*, *funders and faculty alike are giving to interdisciplinarity-and the intensity of the debates that attention has generated- is striking given the fact that relatively little research on many of the underlying issues has been conducted*.*’*

The lack of univocal results on the benefits of IDR stems from the multiplicity of possible perspectives (and ambiguity) on both the concept of interdisciplinarity and the variety of benefits potentially derived from it [13]. In particular, while it is generally observed that research with socio-economic impact is interdisciplinary, the reverse does not hold: there is plenty of interdisciplinary research that is not socially relevant [14,15].

While we acknowledge that IDR may lead to different types of benefits, here we focus our attention to internal scientific dynamics, looking into the relationship between IDR and perceived scientific importance of scholarly contributions, which is proxied by citation impact. Thus we investigate the relationship between citation impact of a publication and its degree of IDR using bibliometric methods. Following established methodology, citation impact can be operationalised in terms of number of citations after conventional field-normalisation [16].

However, the bibliometric operationalisation of IDR remains contentious [13,17,18]. Here we adopt the conceptualisation of IDR as the diversity of disciplinary categories cited in a publication [19,20]. The novelty of this article is that instead of using a single indicator of IDR, we investigate separately how each of the attributes of diversity—namely: *variety*, *balance* and *disparity* of disciplinary categories [21]- affects the citation impact of a publication. The evidence obtained from regression analysis shows that effects of IDR are ambiguous (i.e. they depend on specific choice of diversity): each of the attributes of diversity has a different effect on citation impact. The results indicate that citation impact of publications is positively related with variety, but negatively related with balance and disparity. These results suggest that papers with a *clear disciplinary focus and a small proportions of references* to many proximal disciplinary categories, are comparatively more cited. There is thus no simple relation between IDR and citation impact.

The paper is organized as follows. Section 2 discusses benefits and costs associated with IDR. Section 3 presents a review of the literature exploring with the relationship between IDR and citation impact. Section 4 introduces the conceptualization of interdisciplinary research used in this study. In section 5 the data, measures and methods are described. Section 6 contains the results, which are discussed in section 7. Section 8 presents the conclusions.

## Benefits and Costs of Interdisciplinary Research (IDR)

### Benefits

An ample literature discusses the potential benefits of interdisciplinarity, although most often from a ‘normative and speculative’ rather than analytical perspectives [22]. First, IDR is seen as a source of creativity and innovativeness. Thus, it is beneficial because it generates ‘new research avenues’ and ‘rejuvenates’ the landscape of science. From an evolutionary and ecological understanding of the science system, IDR is a key mechanism to create the recombinations necessary for the system to evolve [23,24].

Second, it is generally argued that IDR is more successful at ‘problem solving’: most scientific puzzles do not fit into disciplinary silos but are best tackled by combining diverse epistemic approaches. Scott Page [12] provides a sophisticated theoretical argumentation on why ‘diversity trumps ability’, i.e. why the combination of diverse perspective, interpretations, heuristics and/or models is better than ‘excellent’ but narrow skills at problem-solving. Building on insights from science and technology studies, Stirling [21,25] also argues that solving complex social problems is best achieved via cognitive diversity, which helps in hedging against ignorance (e.g. unexpected ‘unknowns’), mitigating socio-technical lock-ins, and accommodating plural perspectives. This rationale for IDR is thus particularly strong and convincing in scientific programmes addressing grand societal issues or challenges, such as climate change, epidemic disease, preservation of biodiversity, or innovation-led economic growth, etc., which have become more salient with increasing accountability of science [26,27]. In the case of grand challenges such as AIDS, there is often a plea to bridge the large gaps between distant disciplines such as biomedical research and anthropology (what we will call distal interdisciplinarity), as illustrated by Abdool Karim [28] (p. 31):

‘An underlying obstacle to finding effective ways to intervene is the separation between biomedical and behavioural research in HIV/AIDS. This emanates not only from our failure, as researchers, funders and clinicians, to fully appreciate that every biomedical prevention strategy includes a behavioural change, but also from counterproductive hierarchies and territorialism within science. If behavioural and biomedical scientist work together to develop solutions, the coming decade may prove to be the one when the tide was turned against the global AIDS epidemic.’

Empirical studies support this link between societal problem solving and interdisciplinary research. Van Rijnsoever and Hessels [29] report more propensity for IDR collaborations in researchers that (i) have experience outside academia, and (ii) work in strategic rather than basic disciplines (i.e. in the Pasteur quadrant of fundamental research associated with visions of applications). Similarly, Carayol and Thi [30] provide evidence of a strong association between degree of IDR and industrial links (either collaborations or contractual). Third, Barry et al. [31] (p. 29) argue that this dynamics does not always result only from integration of hitherto unconnected fields but that IDR also ‘springs from a self-conscious dialogue with, criticism of or opposition to the intellectual, ethical or political limits of established disciplines, or the status of academic research in general’. In other words, IDR is born out of intentional struggles for ‘broadening perspectives’ and it is thus seen a source of pluralism [32–34]

### Costs

In spite of the benefits described above, it is now widely acknowledged that conducting IDR entails important efforts, which hinder the chances of success and we will call metaphorically ‘costs’, following Katz and Martin [35]. Two main types of costs can be distinguished: those associated with coordination (or ‘transaction’) and those associated with lack of appreciation of IDR by relevant audiences.

Coordination costs result from the difficulties of integration and are common in team management or collaborations [36,37]. Though IDR does not necessarily entail diverse teams or collaborations, it often does [18]. Coordination costs include: efforts to overcome the lack of a common language, shared meanings and norms within diverse teams; negotiations to harmonize differences in the management and organisational cultures of the collaborating organisations (e.g. on rules of graduate student exchange); administrative load and time needed to manage ‘distributed’ research; expenses to travel over geographical distance.

On the other hand, the social structure of science puts IDR at a disadvantage with regards to the appreciation of the value of interdisciplinary research. This is mainly due to the institutionalisation of science in terms of disciplines. By definition, the function of disciplines is to promote the 'gold standards' in a field and to suppress or marginalise methods, objects and concepts that do not abide to these standards [31]. In spite of the pro-IDR rhetoric of science policy, the norms and rules that govern the scientific enterprise in the everyday management of universities, conferences, recruitment, journals and peer-review favours mono-disciplinary approaches. Turner [22] attributes the institutional dominance of disciplines to the labour-market structure, whereupon PhD granting departments, disciplinary association meetings and undergraduate teaching generate a self-reproductive pattern. Abbott [38] adds to this argument, the intellectual advantage of the main (abstract) disciplines of creating ‘problem-portable’ knowledge, i.e. knowledge that can be re-used for a variety of problems. Bruce et al. [39] reported the following institutional costs from interviews on IDR collaboration: poor career structures for academic interdisciplinary researchers; low esteem by colleagues; difficulty to publish in high ranking journals; discrimination by reviewers in proposals.

Bias in evaluation is another major concern of researchers conducting IDR. This is a topic that has received considerable attention (see monographic issue of *Research Evaluation*, edited and introduced by Laudel and Origgi in 2006 [40], and a literature review by Klein [41]; also Rafols et al. [24] for quantitative evidence). That evaluation of IDR is problematic should not be a surprise. Any evaluation needs to take place over established standards. These standards can be defined within a discipline, but what standards should be used for IDR? A variety of studies have found that what happens, even in the case of multidisciplinary panels, is that IDR ends up being assessed on disciplinary perspectives [42].

The discussion above suggests that IDR benefits are eminently epistemological (i.e. better ways of solving problems, challenging established approaches and nurturing the creation of new knowledge), whilst we can locate the costs in the social sphere (coordination costs) and in the conflicts with disciplinary-based norms (institutional barriers). The extent to which the costs of IDR outweigh the benefits is a matter of open debate and empirical research. Some authors, such as Llerena and Meyer-Krahmer [43] and Cumming and Kiesler [36] have suggested that there is an inverted-U shape relationship between IDR and citation impact: conducting IDR may improve of contribution to knowledge up to a given threshold beyond which further levels of IDR may entail too high coordination costs or institutional barriers. In the following section we review the empirical evidence on the relationship between IDR and citation impact, to shed some light on this matter.

### Evidence on the relation between interdisciplinary and citation impact

The proxy of scientific impact we use here, citations, is a sensible proxy of impact within science, but a problematic indicator for the three broad benefits of IDR discussed above. Citations do not capture opening up new research avenues as often heterodox approaches are peripheral and lowly cited. Moreover, some performance indicators based on citations may underestimate the value of applied research within one field [44]. Finally, although it was widely believed that highly cited is associated with innovativeness, a recent questionnaire by Ioannidis et al. [45], shows that biomedical authors relate their most highly cited publications more to "continuous progress" and "greater synthesis", rather than to "disruptive innovativeness" and "surprise". In summary, one should be very cautious in assuming that higher citations may reflect benefits of IDR.

Several studies have analyzed the relationship between interdisciplinarity and citation impact using different methods and levels of analysis (mainly either at the article or journal level) [46–48]. The most common data source has been the Web of Science (WoS), and the WoS categories (known as ISI Subject Categories up to WoS version 4) have been the most frequently used disciplinary classification [4,24,46].

However, these previous studies did not lead to a consensus regarding the effects of interdisciplinarity on citation impact. Most of the studies rely in relative citation indicators, normalizing the citation counts by field and age of the publications. However they differ in the operationalisation of IDR, most of them based on diversity measures (see Rafols and Meyer, [20] for a review; c.f. Wagner et al. [18]). Here we review some of the most prominent studies, as summarized in Table 1.

**Table 1:** Previous studies on the relationship between IDR and citation impact.

	Steele & Stier (2000) [49]	Rinia et. al (2001) [46]	Adams et. al. (2007) [50]	Levitt & Thelwall (2008) [47]	Larivière & Gingras (2010) [48]	Uzzi & al. (2013) [51]	Larivière & al. (2015) [52]
**Sample**	750 articles in forestry (1985–1994)	All academic groups in physics the Netherlands	Articles from two UK universities	All science and social science articles	All papers published in WoS in 2000	All papers in WoS (1990–2000)	All papers in WoS (2000–2012)
**Database**	*Journal Forest Science*	WoS	WoS	WoS and Scopus	WoS	WoS	WoS
**Unit of analysis**	Article	Journal	Article	Journal	Article	Article	Co-citation
**IDR Indicator**	Brillouin’s diversity index	% papers not published in physics	Shannon diversity & % cited refs. to other SC	Number of disciplines assigned to journals	% cited refs. to other SC	Median disparity, 10% percentile disparity	Dichotomous: Intra. vs. Inter subsdiscipline
**Aspect of diversity**	Combination of variety and balance	Balance	Combination of variety and balance	Variety	Balance	Disparity	Disparity
**Measure of citation impact**	Average annual citation rate	Normalized indicators	Normalized indicators	Normalized indicators	Normalized indicators	Not normalized	Normalised indicators
**Correlation IDR vs Impact**	Positive	No effect	Visual evidence of inverted U	Negative effect in some disciplines	Inverted U shape	Low median disparity, with high 10% disparity	Mainly positive
**Regression controls**	Yes	No	No	No	No	Yes	No

Steele and Stier [49] estimated the degree of interdisciplinarity applying Brillouin’s diversity index (related to Shannon’s entropy) to the disciplinary categories of references in an article and they found a positive and significant effect of IDR on the citation impact. Rinia et al. [46] found no significant correlation in a study on physics between the degree of interdisciplinarity and citation impact, measuring the degree of interdisciplinarity as the proportion of papers published by physicists in disciplines other than physics. A report by Adams et al. [50] explored the relation between interdisciplinarity (operationalised as the Shannon entropy of disciplinary categories in the references of articles) and citation impact (measured by the number of citations received by papers), and did not report a systematic association between the most interdisciplinary papers and the amount of citations received. However, they suggested from visual inspection that the articles with highest citation rates scored intermediate levels of interdisciplinarity, implying an inverted U-shape relationship between interdisciplinarity and citation impact.

Levitt and Thelwal [47] found that number of citation to multidisciplinary journals (those related to more than one disciplinary category in the database) were roughly 50% less than monodisciplinary articles. This correlation was found using Scopus as data source and only for a limited number of disciplines in the natural sciences. When the analysis was focused on the social sciences neither in Scopus nor WoS were significant correlations found between the level of interdisciplinarity and the citation impact.

A study conducted by Larivière and Gingras [48] analysing all articles included in the WoS in 2000, did not find a clear correlation between the proportion of citations to other disciplines (their indicator of interdisciplinarity) and the citations received. The key finding of these authors was that, in all disciplines, highly disciplinary or highly interdisciplinary were associated with a low citation rate, suggesting an inverted U relationship between citation impact and interdisciplinarity.

A study by Uzzi et al. [51] investigated the effect of conventional and atypical reference combinations in the citation impact of a publication. Conventional reference combinations are co-citations of journals that are often co-cited and hence proximate in cognitive space (e.g. *Scientometrics* and *Journal of Informetrics*), whereas atypical combinations are those that are distant in cognitive space [53]. Therefore, the study can also be interpreted as exploring the relationship between type of interdisciplinarity and citation impact. Uzzi et al. [51] found that the probability of a publication being highly cited was significantly higher for papers that make mostly conventional combinations of journals (i.e. that cite similar journals), but which have a small proportion of atypical combinations (i.e. that cite just a few disparate journals). Hence this study also suggests that there is not a simple relationship between degree of IDR and citations, and supports the hypothesis that middle ground in IDR is most conducive to high number of citations.

Recently Larivière et al. [52] have analysed the citation impact of interdisciplinary publications, looking at the effect of interdisciplinary co-citations on the citation impact of the citing publications. IDR is thus a dichotomous variable: either a co-citation is intra- (same discipline) or interdisciplinary. They find that most interdisciplinary combinations have a positive effect on citation impact, which increases with cognitive disparity. The interpretation (and comparison with previous work) of this study with IDR practices is difficult given that its unit of analysis is the co-citation of categories of references, rather than the article or the research group.

Another choice in Larivière et al.’s study that differs from previous approaches, is that cognitive distance is computed over a cylindrical projection in a 2 dimensional map–instead of using the direct cognitive distance derived from the multidimensional space of 554 subdisciplines. Using the 2 dimensional projection to compute cognitive distance appears to work better than direct cosine distances for highly dimensional spaces, for example in journal maps [54], but may result in some artefacts.

## A Multidimensional Conceptualisation of IDR: Variety, Balance and Disparity

The focused literature review presented above shows the variety of indicators used to measure the notion of interdisciplinary research and their limited capacity to obtain comparable findings. We propose that the lack of agreement is partially due to the assumption implicit in previous studies that the concept of interdisciplinarity is a mono-dimensional property. Here we aim to carry out a more fine grained study by understanding interdisciplinarity as diversity of disciplinary categories, and then analysing separately the effect of the different aspects of diversity, namely: variety, balance and disparity [21,24,55].

**Fig 1:**
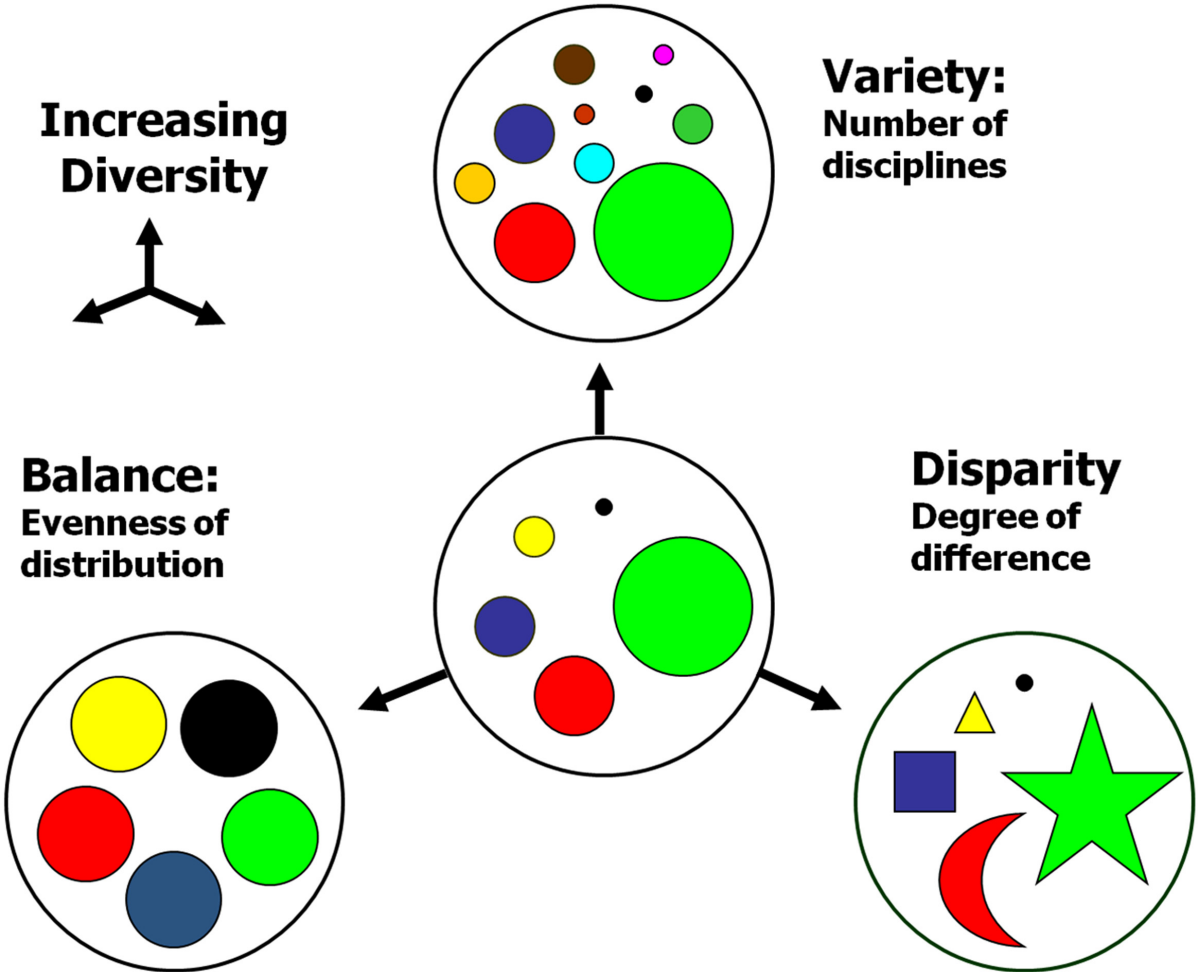
Schematic representation of the attributes of diversity, based on Stirling [25].

Here we adopt a definition of interdisciplinarity based on the concept of integration: a mode of research that *integrates* concepts or theories, tools or techniques, information or data from different bodies of knowledge [2,4]. In order to capture the process of integration, i.e. the process in which previously different and disconnected bodies of research become related, we rely on the concept of diversity as proposed by Stirling [21] and illustrated in Fig 1. This concept refers to three different attributes of a system comprising different categories: (i) Variety: number of distinctive categories; (ii) Balance: evenness of the distribution of categories; (iii) Disparity or similarity: degree to which the categories are different/similar. An increase in any of these attributes results in an increase in the diversity of the examined system.

Indicators aiming at capturing the degree of diversity in studies of interdisciplinarity (i.e. disciplinary diversity) rely on the established disciplinary classifications so that *variety* generally refers to the number of disciplinary categories, *balance* is related to the evenness of the distribution of disciplines and *disparity* measures the extent of which these disciplines are different/similar from a cognitive point of view.

We have calculated these three different aspects of disciplinary diversity as indicated in Table 2. The creation of distinct variables representing "purified" attributes of diversity is a tool to explore the different influence of the attributes. However, one should handle very careful these "purified" variables, as they may misrepresent diversity. For example, if on adopts a classification with some fine grained classes (Japanese literature and Finnish literature) and some coarse grained classes (Life Sciences), indicators of variety and balance will be meaningless unless disparity is taken into account. It is in this sense that [21] (pp. 709–710) explains that the three properties of diversity are co-constituted.

**Table 2:** Operationalisations of the attributes of diversity.

Attribute of diversity	Operationalisation*
**Variety**	We use the number of distinctive WoS categories (n) cited in an article.
**Balance**	We use Shannon diversity (H) normalised by variety (n), where *p*_*i*_ is the proportion of references in WoS category *i*:$\text{Balance}=-\frac{1}{\text{ln}(n)}\sum_{i}p_{i}\text{ln}p_{i}$
**Disparity**	We use a measure of disparity is based on the average cognitive distance between WoS categories within the reference list. The cognitive distance between two disciplines is calculated as *d*_*ij*_ = 1-*s*_*ij*_, with *s*_*ij*_ being the cosine similarity between each pair of disciplines *i* and *j*. The sum is over disciplines with at least one cited reference: $\text{Disparity}=\frac{1}{n(n-1)}\sum_{ij}d_{ij}$

* Note: Many other operationalisations of these properties are possible. For example, we could have taken n^2^ instead of n as variety, or the median disparity rather than the mean disparity of a reference set.

The operationalisation of these three different indicators aims at capturing and isolating each dimension of diversity. This approach enables us to analyse whether or not these individual attributes provide distinctive insight about diversity, and also to examine if they have a distinct influence on citation impact. However, one has to keep in mind that all measures of diversity are highly dependent on the classification used and the associated metrics. We also must caution the readers that isolated measures of variety, balance and disparity are more likely to produce artefacts than integrated measures such as Rao-Stirling.

## Data and Methods

### Data

We collected articles (i.e. only document type "article", not including others such as reviews, letters, etc.) published in 2005 from Science Citation Index-Expanded (SCI-E) belonging to four WoS categories i.e. Cell Biology (CBIOL), Engineering, Electrical & Electronic (EEE), Food Science and Technology (FSTA) and Physics, Atomic, Molecular & Chemical (Physics-AMC). These four fields cover applied research (i.e. engineering and food science) and basic research (i.e. physics and cell biology). The total number of collected papers amounts to 72,116 records (CBIOL n = 16,922; EEE n = 30,574; FS&T n = 10,869; Physics-AMC n = 13,751). In order to estimate the disciplinary background of a paper we considered that it would be necessary to have a minimum of four references linked to a WoS subject category. Given the multi-assignation a unique reference may be linked to more than one WoS Category. Hence we removed from our sample those papers below this threshold. The total amount of deleted papers was 9,708 (CBIOL n = 161; EEE n = 8,351; FS&T n = 832; Physics-AMC n = 364), thus our final dataset comprises 62,408 papers.

### Measures

#### Dependent Variable

We have measured citation impact in terms of normalized number of citations. We calculated the Normalized Citation Score (NCS) for each publication, which compares the number of citations of each publication with the average number of citations of all publications in the same WoS category and in the same year [56], using a fixed citation window of five years.

It is important to note that the distribution of citations per article is skewed. About 10% of the 62,408 (i.e. 6,107) articles in our sample did not receive any citation and 50% received less than 7 citations (with a maximum of 782 citations). Median of citations per paper vary among disciplines (12 in CBIOL, 6 in Physics-AMC, 5 in FS&T and 4 in EEE) as well as percentages of not-cited articles (15.42% in EEE, 10.52% in FS&T, 7.75% in Physics-AMC and only 3.5% in CBIOL). In order to attenuate the skewed distribution of this variable, we have used a natural logarithm transformation of our proxy of scientific impact, after having added 1 to retain the zeros. Our dependent variable is labelled: *ln (NCS)*.

#### Independent variables: variety, balance and disparity

In order to calculate disciplinary diversity, we consider WoS categories related to the reference list in a given paper. Our assumption is that the citing paper *integrates* knowledge from the WoS categories to which the cited papers belong. In order to operationalize this idea, we considered the distribution of WoS categories in the references cited by the papers in our sample. We obtained the distribution of WoS categories by transforming the list of journals in which the references were published into a list of WoS categories according to the Journal Citation Reports.

Table 3 presents some statistics on the number of papers, references and linked references to WoS-categories for our final sample.

**Table 3:** Description of final sample, broken down by field of science.

WoS Category	Papers	References	Median	Mean±SD	% Linked refs
**CBIOL**	16,761	701,832	40	41.87±17.14	93.32%
**EEE**	22,223	447,660	17	20.14±12.12	55.23%
**FS&T**	10,037	284,069	26	28.30±14.27	74.41%
**Physics-AMC**	13,387	435,101	29	32.50±17.82	81.25%
**Total**	62,408	1,868,662	26	29.94±17.50	78.51%

After deleting those articles with fewer than four references linked to WoS categories, the final dataset of 62,408 articles citing 1,868,662 references, and the overall share of references linked at least to one WoS Category is 78.51%. This can be considered a high percentage if compared to the findings of Lariviere and Gingras [48], who found the highest scores of cited references linked to WoS categories in medical fields (around 79%).

The distribution of WoS categories in the reference list allowed us to compute *variety*, *balance* and *disparity* as described in section 4: *variety* as the number of WoS categories (n) that appeared at least once and *balance* as the evenness of the distribution of WoS categories. In order to compute the *disparity* measure, a similarity matrix s_*ij*_ for the WoS categories must be constructed. To do so, we created a matrix of citation flows matrix between WoS categories, and then converted it into a Salton’s cosine similarity matrix in the citing dimension. The s_*ij*_ describes the similarity in the citing patterns for each pair of WoS categories in 2006, for the SCI set (175 WoS categories). A detailed description and analysis of this s_*ij*_ SC-similarity matrix is provided elsewhere when describing global maps of science [20]. See descriptive statistics for all these variables in the Table 4 below.

**Table 4:** Descriptive statistics (number of observations: 62,408).

	Average	St.Dev.	Median	Minimum	Maximum
ln (NCS)	0.554	0.471	0.455	0.000	4.777
Variety*	0.227	0.144	0.212	0.000	1.000
Balance	0.812	0.141	0.835	0.000	1.000
Disparity	0.581	0.149	0.598	0.024	1.000
Rao-Stirling	0.367	0.148	0.372	0.000	0.804
n_authors	4.232	2.719	4.000	0.000	226.000
n_inst	2.062	1.284	2.000	0.000	38.000
National_collab	0.381	0.486	0.000	0.000	1.000
Internat_collab	0.212	0.409	0.000	0.000	1.000

* While the original values of Variety range between 1 and 34, this variable has been transformed to range within the 0–1 interval, in order to build it similar to balance and disparity.

We have transformed the variable Variety as follows: Variety_i_ = (Y_i_−Y_min_) / (Y_max_−Y_min_), where Y_min_ and Y_max_ are the extreme values of the original variable Y.

Finally, we have also included in our analysis an indicator of diversity that incorporates the three aspects of diversity (variety, balance and disparity) in a single measure: i.e. Rao-Stirling [20,21]. The Rao-Stirling diversity indicator can be expressed as follows:

$$\text{Rao-Stirling diversity}=\sum_{ij}^{i}p_{i}p_{j}d_{ij}$$

See Zhang et al [57] for a recent re-formulation (not used here) of the Rao-Stirling diversity that might improve its sensitivity to high values of diversity. We explicitly consider this indicator for the purpose of having a benchmark for comparison, with regards to the separate impact of the three aspects of diversity.

#### Control variables

We have included a number of control variables that the literature has considered as potentially associated with the number of citations received by scientific publications [58–60]. First, we control for the number of authors (*n_authors*) and the number of institutions in the publication (*n_inst*), since these features have been repeatedly found to be associated with the number of citations received by publications. Second, we have controlled for the geographic scope of institutional collaboration by building a set of three dummy variables. *National_collab* takes value 1 if there are at least two different institutions from the same country. *Internat_collab* takes value 1 if the paper has been produced in collaboration between two or more different countries. And *No_Collab* that takes value 1 if only one institution participates in the paper. These three binary variables are aimed to capture whether publications involving an international collaboration are positively associated with citation impact (compared to publications involving either domestic collaboration or no collaboration). Third, we have constructed a dichotomous variable to control for the four WoS categories considered in this analysis (i.e. CBIOL, EEE, FSTA or Physics-AMC). These discipline-level controls are important to account for field-specific citation patterns that may influence the relationships estimated between our three aspects of IDR and citation impact. Finally, based on authors’ affiliation addresses, we also included country-level dummies to account for the effect of particular countries in the citations received by publications (this includes dichotomous variables for affiliation addresses corresponding to: China, France, Germany, Japan, South Korea, Spain, UK and US).

In summary, we control for variables that represent social aspects of the research input (number of authors and institutions, national and international collaborations, discipline, country), and that may have an effect both on the citations received and on degree of IDR. For example, the number of authors may be associated with higher citation impact and higher interdisciplinarity. However, we do not control for variables such as number of references or pages that reflect the characteristics of the research output (i.e. the paper) even if they are known to be related to interdisciplinarity [61], as these choices are made by authors in order to express (rather than to construct) the interdisciplinarity of the research. For example, a larger number of pages or references in a paper may reflect the need of interdisciplinary in the contents.

Nevertheless, for the sake of robustness, we have also controlled for the number of references in papers. This control is reasonable since all our three constructs of IDR (variety, balance and disparity) are based on the references cited in the papers; but it is also problematic, because the total number of references in a publication is extremely highly correlated with the measure of variety (a Pearson correlation of 0.60). In order to avoid problems of multicollinearity between the variables ‘number of references’ and the three measures of IDR, we have built two dichotomous variables. The first one takes value 1 for all those publications that belong to the bottom quartile in terms of number of references: thus, we control for publications with low number of references (i.e. those publications that have 17 or less references, accounting for 25% of publications in our sample: N_refer_small). The second variable takes value 1 for those publications that belong to the top quartile in terms of number of publications (i.e. those publications with 39 or more references, which account for the 25% of publications in our sample with the largest number of references: N_refer_large). The estimates of our regression analysis including these controls are shown in the Table A in the Supporting Information File (S1 File), and indicate that the sign and statistical significance of results regarding the effects of variety, balance and disparity are largely aligned with the results presented in section 6.

Since our dependent variable (log transformed of Normalized Citation Score, ln (NCS)) is a continuous variable with a lower boundary at zero and a upper boundary at infinity, and a significant proportion of the observations in our sample are zeros (i.e. about 10% of publications receive no citations), we have used a Tobit regression model to account for the disproportionate number of observations with zero values, and avoid inconsistent estimates from Ordinary Least Square (OLS) regression.

Table 4 provides the descriptive statistics and Table 5 the correlation matrix for all the variables used in the analysis. Table 5 shows that the correlations between our independent variables are rather low: we find positive correlations between variety and balance (i.e. 0.15) and between variety and disparity (0.19), and a negative correlation between balance and disparity (-0.23). These results provide a first descriptive evidence that these three measures of diversity reflect different properties of interdisciplinarity, and are worth considering separately rather than brought together in a single index. We will next examine to what extent these three attributes of diversity have a distinct effect on citation impact.

**Table 5:** Correlation matrix.

	ln(NCS)	Variety	Balance	Disparity	Rao-St.	n_auth.	n_inst.	Inter_coll.
Variety	0.070*							
Balance	-0.053*	0.148*						
Disparity	0.019*	0.185*	-0.222*					
Rao-Stirling	-0.007	0.483*	0.518*	0.580*				
n_authors	0.087*	0.222*	0.048*	-0.065*	0.034*			
n_inst.	0.095*	0.200*	0.030*	-0.016*	0.060*	0.590*		
Inter_collab	-0.076*	0.037*	-0.011*	0.010*	0.009*	0.218*	0.256*0.679*	
Nat_collab	0.002	0.112*	0.030*	-0.008*	0.045*	0.179*	0.342*	-0.407*

* *p* < 0.05

## Results of Regression Analysis

This section reports the results of our analysis about the effects of interdisciplinary research on citation impact. Table 6 reports the results of Tobit estimates for the whole sample (i.e. 62,408 observations). We present the results in six columns: the first two columns display the results for the relationship between a full indicator of IDR (Rao-Stirling diversity), and citation impact. Column (3) shows the linear effects of each of the diversity measures on our normalized measure of citation impact, while the remaining three columns—columns 4 to 6—display results regarding evidence of a curvilinear relationship between diversity measures and citation impact, by introducing the quadratic term for each of the diversity measures in turn.

**Table 6:** Tobit estimates for the effect of variety, balance and disparity on citation impact.

	Dependent variable: *ln(Normalized Citation Score)*					
Variables	(1)	(2)	(3)	(4)	(5)	(6)
Rao-Stirling	-0.006	-0.027				
	(0.014)	(0.054)				
Rao-Stirling^2^	---	0.029				
		(0.074)				
Variety	---	---	0.552 ***	1.437 ***	0.463 ***	0.542 ***
			(0.019)	(0.050)	(0.020)	(0.019)
Balance	---	---	-0.326 ***	-0.385 ***	0.811 ***	-0.360 ***
			(0.016)	(0.016)	(0.054)	(0.017)
Disparity	---	---	-0.163 ***	-0.198 ***	-0.043 **	0.183 **
			(0.017)	(0.017)	(0.017)	(0.072)
Variety^2^		---	---	-1.395 ***	---	---
				(0.074)		
Balance^2^	---	---	---	---	-0.915 ***	---
					(0.041)	
Disparity^2^	---	---	---	---	---	-0.317 ***
						(0.064)
N_authors	0.0 ***	0.015 ***	0.014 ***	0.014 ***	0.015 ***	0.014 ***
	(0.001)	(0.001)	(0.001)	(0.001)	(0.001)	(0.001)
N_Institutions	0.013 ***	0.013 ***	0.011 ***	0.011 ***	0.011 ***	0.011 ***
	(0.003)	(0.003)	(0.003)	(0.003)	(0.003)	(0.003)
Internat_collab	0.017 **	0.017 **	0.016 **	0.014 *	0.014 *	0.016 **
	(0.007)	(0.007)	(0.007)	(0.007)	(0.007)	(0.007)
National_collab	0.007	0.007	0.005	0.003	0.006	0.005
	(0.006)	(0.006)	(0.006)	(0.006)	(0.006)	(0.006)
CBiol	-0.037 ***	-0.037 ***	-0.129 ***	-0.136 ***	-0.110 ***	-0.128 ***
	(0.006)	(0.006)	(0.007)	(0.007)	(0.007)	(0.007)
EEE	0.029 ***	0.029 ***	0.061 ***	0.089 ***	0.085 ***	0.067 ***
	(0.006)	(0.006)	(0.006)	(0.006)	(0.006)	(0.006)
FST	-0.003	-0.003	-0.025 ***	-0.021 ***	-0.018 ***	-0.022 ***
	(0.007)	(0.007)	(0.007)	(0.007)	(0.007)	(0.007)
Constant	0.378 ***	0.381 ***	0.634 ***	0.595 ***	0.266 ***	0.575 ***
	(0.008)	(0.011)	(0.018)	(0.018)	(0.025)	(0.022)
N. obs.	62408	62408	62408	62408	62408	62408
Log-Likelihood	-47415.647	-47415.568	-46908.8	-46729.1	-46663.5	-46896.7
LR χ^2^	2699.95***	2700.1***	3713.7 *** ***	4073.0 ***	4204.1***	3737.9***

Notes:

* *p* < 0.1;

** *p* < 0.05;

*** *p* < 0.01.

Standard errors are in parenthesis.

Eight dummies have been included in the regression to account for the effect of countries (from the authors’ affiliations) in the number of citations received.

These dummies are not reported in the Table.

First, Table 6 shows that there is no evidence of a statistically significant relationship, either positive or negative, between the composite indicator of IDR (Rao-Stirling diversity) and citation impact. This finding runs apparently contrary to the presumption that IDR has a significant impact on citations. Given the non-significant outcome of Rao-Stirling (which is a distance weighted Simpson index), we also investigated the effect of a distance weighted Shannon diversity, with a similar non-significant results.

However, Column (3) in Table 6 shows that the three aspects of diversity have a statistically significant and distinct effect on citation impact. While variety is positively associated with citation impact, balance and disparity are negatively associated with citation impact. Therefore, the number of different WoS categories a publication draws upon has a strong positive effect on the citation impact, but this effect can be outweighed by the effects of too high a distance between the WoS ategories (high disparity) or too even a distribution across WoS categories (high balance).

**Fig 2:**
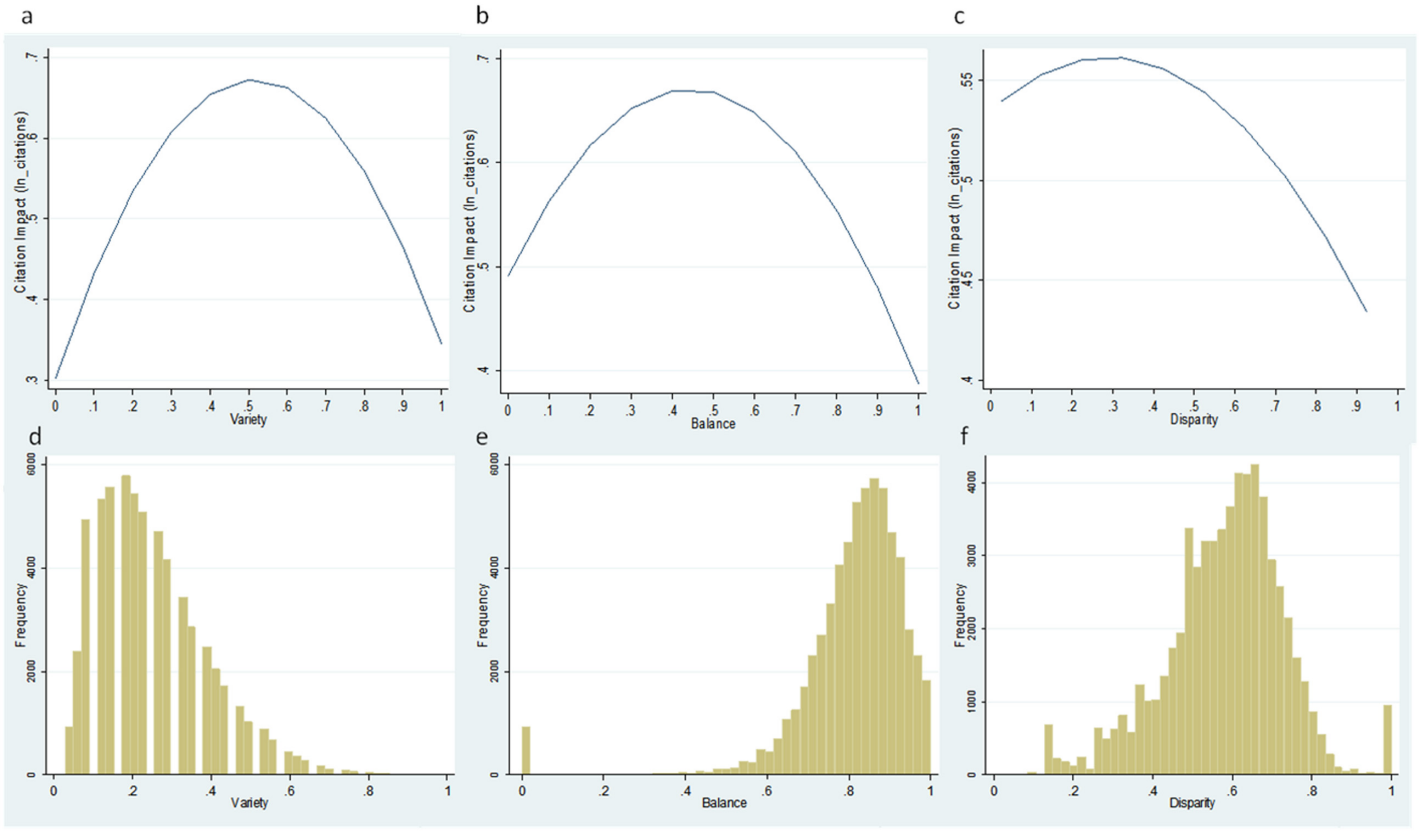
Top: Relation between variety (a), balance (b) and disparity (c) with citation impact as found in the regression analysis. Bottom: Distribution of articles with over variety (a), balance (b) and disparity (c).

The second important result from Table 6 is that all the quadratic terms are statistically significant and negative. For all three diversity measures, the results from Table 6 indicate the presence of a curvilinear inverted U-shape between each of the separate diversity measures and the citation impact of publications. This curvilinear relationship indicates, in principle, that while variety, balance and disparity have an initial positive effect on the citation impact of publications, a threshold is reached beyond which higher levels of diversity might be detrimental to the citation impact of publications. This curvilinear relationship is illustrated in Fig 2, showing the inverted U-shape relationship for each of the three aspects of diversity.

We replicated the analysis for our four WoS categories: Cellular Biology (CBiol), Electrical and Electronic Engineering (EEE), Physics (PHY) and Food Science and Technologies (FST). These results are overall consistent with those obtained for the complete sample. In particular, we observe that the three aspects of diversity have all a significant effect on the citation impact of publications, and with a similar sign to that obtained for complete sample (with minor exceptions). Moreover, we also observe that the curvilinear inverted U-shape relationship does generally apply for most of the cases in which a quadratic term is introduced in the regression analysis. These results have not been included in the paper but are available from request to the authors.

However, a more careful inspection of Fig 2 reveals that the distributions of articles in the curvilinear relationships in the inverted U curves, fall in the positive side of the slope for variety and the negative side of slopes for balance and disparity. This means that most articles would increase their citations by increasing variety and by decreasing balance and disparity—in agreement with the linear model in column (3).

Regarding other determinants of citation impact included in the analysis, our findings are consistent with results in previous studies. We have found that citation impact is positively and significantly shaped by: the number of authors and the number of institutions involved in a paper. We have also found support for the positive impact of international collaborations on the citations received by a paper, even though this effect is in some cases weakly statistically significant (in agreement with the review by Frenken et al. [62]).

## Discussion

In this paper we have investigated the relationship between interdisciplinary research and citation impact. A key novel element in our study is the way in which we operationalise the concept of interdisciplinarity by exploring separately the three different attributes of diversity, i.e. variety, balance and disparity. This more comprehensive implementation of interdisciplinarity accounts not only for the dimensions of variety and balance but, unlike previous studies, also encompasses cognitive distance, i.e. disparity. Our results show that the relationship between interdisciplinary research and citation impact is heavily dependent on how IDR is measured and operationalised. Another difference of our study with previous approaches is the use of multivariate regression analysis. This allows us to disentangle the effect of our three measures of IDR on citation impact, once accounting for the effects of a wide range of control variables.

The first contribution of this study is that different aspects of diversity push in distinct and possibly opposite directions when examining their association to citation impact. These distinct effects of the various components of diversity are likely to be the reason for the contrasting findings in the literature, which has pointed out in all possible directions: positive, negative and curvilinear relationships between IDR and citation impact. These different effects, may also explain why the full indicator of IDR (i.e. Rao-Stirling diversity), which expresses the three aspects of diversity within a single measure, shows no statistically significant association with citation impact. However, in contrast with this result, we find that the three aspects of diversity have a strong significant effect when they are examined as independent explanatory factors: variety is positively associated with citation impact, while balance and disparity are negatively associated with citation impact.

Our results further qualify the separate effect of the three aspects of diversity by pointing out that all three dimensions of IDR (i.e. variety, balance and disparity) display a curvilinear relationship with citation impact. In other words, there is an inverted U-Shape relationship between citations received and the number of WoS categories cited (variety), the distribution of references over WoS categories (balance) and the cognitive distance of the references (disparity). This means that there is a threshold beyond which more of any of the different aspects of IDR may be detrimental to citation impact. However, despite of evidence supporting an inverted U-shape curvilinear relationship, it is important to highlight that the bulk of publications are located along the upward side (below “optimum”) of the curvilinear relationship between variety and citation impact; while instead, the large majority of the publications in our sample concentrate on the downward side (above “optimum”) of the curvilinear relationship between balance or disparity and citation impact (Fig 2).

The negative effect for disparity we find is at odds with the recent report by Larivière et al. [52] that disparate IDR leads to higher citation impact. The disagreement may have various origins. First, since Larivière et al.'s findings are not based in a regression, the difference may be due to the fact of not controlling for variables such as type of collaboration or number of authors—indeed, in the correlation analysis shown in Table 3 we also find a positive and significant relationship between citations and disparity which becomes negative once controlling for the effects on citations from other covariates. Second, we notice that Larivière's finding is about impact accrued by referencing combinations, not publications—which is of difficult translation in sociological terms, i.e. it is unclear how it reflects the IDR of a research effort. Moreover this approach does not take into account the proportions of categories referenced within a paper, but only whether two disciplines are co-cited. In doing so, they may be emphasising the contribution of small proportions of references–thus sometimes counting as “distal IDR” what in our approach would be “proximal IDR”. Third, the cognitive distance used by Larivière et al.’s is derived from a two dimensional projection, which might yield some artefacts.

A first insight from these results is that publications that accrue the most citations are moderately interdisciplinary (neither too much, nor too little), in accordance with suggestions from previous studies [48,50]. The key new insight of this study is that highly cited papers tend to cite various disciplinary categories (higher variety), but cite little outside their disciplinary vicinity (lower disparity) and in small proportions (lower balance). We propose the concepts of **proximal** and **distal interdisciplinarity** to interpret these results. Distal interdisciplinarity would refer to bold interdisciplinary papers that draw a significant proportion of references from disparate disciplines. According to various studies this type of work is unlikely to become highly cited. Instead, proximal interdisciplinarity would reflect more cautious research practices that go beyond the immediate sub-discipline, but still mainly draw on related knowledge. Our study, in everyday terms, suggests that practicing ‘meek’ or ‘shy’ (proximal) interdisciplinarity pays off in citations, but that brazen, audacious (distal) interdisciplinary efforts are not rewarded with citation success.

The results should be taken with caution given various limitations. First, the diversity measures used are just one of various possible and equally legitimate measures of variety, balance and disparity. Second, the inaccuracies in the WoS categories used to define subdisciplinary categories may create biases in the indicators of citation impact (since citation impact is highly affected by normalisation [24,44]) and may have an important effect as well in diversity measures. Third, we do not consider potential differences in behaviour between disciplines since the four WoS categories studied show relatively similar results. However, other disciplines might have different dynamics [52]. Fourth, in this study we use a 5-year window that might be insufficient for IDR research, since IDR may accrue citations later and over longer periods. For instance, although variety and disparity have a negative effect on diversity with 3-year windows, they have a positive effect with 13-year windows according to Wang et al. [61]. Sixth, the inclusion or not of some control variables such as number of co-authors, institutions or article length is open to debate and these may have an effect on results.

The differences in field classification, citation window and control variables across studies may explain the sometimes contradictory results found in different studies. A systematic, muldimensional approach testing many hypotheses will be needed to find out which factors from those listed above explain the source of disagreement between the different recent publications. For example, the partial disagreement of our results with Wang et al. [61], which use a similar conceptual framework, might be due to various choices: i) first of all and most importantly, they make complex constructs for variety, balance and disparity, deriving them from factor analyses carried out using composite diversity measures such as Gini, Shannon and Rao-Stirling or number of references (in our view, this makes Wang’s definition of variety, balance and disparity vague and possibly problematic, as it is not fixed on a conceptual basis); ii) they use 3 and 13 year citation windows; iii) they control for paper length and number of references; iv) they control field effects at the journal rather than at the level of WoS category; v) they don’t control for some social aspects such as country or number of institutions which have an influence on citation impact.

Another serious limitation for the policy relevance of this study is that the analysis is based on the IDR of single publications, instead of analysing the degree of IDR in a given research group or project, which would be the proper sociological unit of analysis. We pose the hypothesis that the "optima" of diversity found for papers can be lower than the optima for research groups, since failed and risky interdisciplinary articles may feed fruitful knowledge into future IDR efforts.

These findings may portray two distinct social dynamics. On the one hand, they are consistent with the view that high citation impact research is achieved in scientific efforts clearly positioned in a given field with only a small proportion of contributions from related fields (proximal IDR). This finding aligns with the notion that researchers have bounded rationality and are only capable of making productive knowledge combinations within their cognitive proximity, as distant explorations are associated with high uncertainty [63]. Distal IDR might be highly successful in a few cases, but in average it produces more failures (with lower citation impact) due to coordination costs described in section 2 such as lack of epistemic understanding across partners or bureaucratic hurdles [36,43]. Studies in other areas of knowledge management have found analogous results; for example, innovative performance of firm alliances shows an inverted U-shape dependence on the technological distance between firms [64].

A second interpretation of the findings is that scientific audiences do not have enough absorptive capacity for reading, valuing and then citing unconventional knowledge combinations. Indeed, a questionnaire among highly cited researchers found that many of them did not rate disruptive innovativeness or surprise as the dominant characteristics of their most highly cited papers [45]. According to this view, the problem with distal IDR is not the "value" of IDR contributions, but the incapacity of scientific readers to appreciate atypical research—analogous to the incapacity of art connoisseurs to appreciate Van Gogh's paintings while he was alive because they were too unconventional.

A recent study by Uzzi el al. [51] proposes an alternative interpretation of what a middle ground degree of interdisciplinarity might be. Rather than examining three characteristics of diversity separately, they describe the distribution of disparities between the references within one paper and characterise interdisciplinarity with two variables. First, they create a measure of a paper diversity with the median disparity between references within an article (very similar to Rao-Stirling's diversity used here, which is the mean disparity). Since in our system the disparity distributions are normally distributed, the mean and the median are very close.

Second, they measure the disparity value for the top 10% percentile, which captures to which extent an article contains atypical combinations of references. They find that highly cited research tends to have a low median disparity and a high top 10% percentile–a result that is also a "middle ground" between the lack of creativity of monodisciplinary research and the risk of highly interdisciplinary approaches. The science dynamics interpretation of Uzzi's findings is compatible with the framework presented in section 2 according to which the benefits of recombinations are weighted against the costs of knowledge integration. A more detailed comparison will be needed to map the relationship between our findings and Uzzi et al.'s approaches, given differences in granularity (WoS categories vs. journals), the distance metrics of disparity (cosine similarity vs. z score) and the disciplines analysed [53].

## Conclusions

This article confirms that the relationship between interdisciplinarity and citation impact is complex. Very low or very high degrees of IDR are found to decrease citation impact, whereas some middle degree of IDR, which we characterised as proximal interdisciplinarity, tends to have higher citation impact. More research is needed to further develop robust characterisations of this middle degree of IDR and compare their predictive capacity, given the similarities and differences between our results and those of other approaches such as Uzzi's [51]. The complexity of the findings and their contrast with some other recent results supports the view that stylised descriptions of science dynamics in terms of Newton-like laws are empirically problematic, as the conclusions depend on technical assumptions such as field classification and control variables that are currently made without a sound theoretical basis. Interpretations leading to simple advice such as “the more interdisciplinarity, the better” may be harmful for policy as they give a false sense of certainty [65].

Our results are consistent with some previous studies in finding that publications with long-distance or distal IDR are not, in average, rewarded with a high citation impact (but they stand apparently in contrast to recent reports by Larivière et al. [52] and Wang et al. [61]). However, this study has focused only on citation impact as a proxy for scientific impact. We believe that future research should also pose the question whether IDR (and particularly distal IDR) might be an important contribution of science for grand challenges or societal problems. For example, Chavarro et al. [55] found that locally relevant knowledge in a developing country such as Colombia tends to be associated with distal IDR (higher balance and disparity, lower variety) rather than with proximal IDR. Hessels et al. [66,67] have empirically documented across various fields the tensions that researchers focused on societal issues experience against when subject to bibliometric evaluations. One can thus speculate of a lack of alignment between reward incentives in academia (citations) and societal needs or demands [68]. Therefore, it remains an open issue whether distal IDR is associated with long-term societal impact of research that is only poorly captured by citations, and to what extent science policy initiatives may be needed to support distal rather than proximal IDR (which may be already supported by citation rewards).

The two alternative interpretations of the findings advanced in the previous section suggest two different and complementary action lines. First, following the logic that distal IDR is more complex and risky, policy actions might be required to reduce coordination and institutional barriers and facilitate the formation of interdisciplinary research teams and projects. Collaboratories, targeted funding and removal of old regulations for field-hopping might be examples of these type of instruments. Second, following the logic that low recognition of distal IDR is due to the difficulties of the research community to adequately value and asses unconventional research, actions would be needed with the longer term goals of changing disciplinary and institutional cultures, such as pluralising editorial boards of journals with higher visibility and supporting interdisciplinary practices in higher education [2]. This speculative discussion thus calls for advancing research that investigates the societal impact of distal interdisciplinary research.

## Supporting Information

S1 File Effect of variety, balance and disparity, controlling for number of references. (DOCX)

## References

[1] Jacobs JA, Frickel S. Interdisciplinarity: a crititical assessment. Annual Review of Sociology. 2009;35: 43–65.

[2] National Academies of Science. Facilitating Interdisciplinary Research. Washington, D.C: National Academies Press; 2004.

[3] Braun T, Schubert A. A quantitative view on the coming of age of Interdisciplinarity in the sciences, 1980–1999. Scientometrics. 2003;58: 183–189.

[4] Porter AL, Roessner JD, Cohen AS, Perreault M. Interdiscipinary research: meaning, metrics and nurture. Research Evaluation. 2006;15: 187–196.

[5] Rhoten D, O’Connor E, Hackett EJ. The Act of Collaborative Creation and the Art of Integrative Creativity: Originality, Disciplinarity and Interdisciplinarity. Thesis Eleven. 2009;96: 83–108.

[6] ERC. ERC Grant Schemes. Guide for Applicants for the Starting Grant 2011 Call. [Internet]. Available: ftp://ftp.cordis.europa.eu/pub/fp7/docs/calls/ideas/l-gfastg-201101_en.pdf.

[7] Bauman Z. Liquid life. Cambridge, UK: Polity; 2005.

[8] Hoffman E. The new nomads In: Aciman A, editor. Letters of transit Reflections on exile, identity, language and loss. New York: The New Press; 1999.

[9] Hollingsworth R, Hollingsworth EJ. Major discoveries and biomedical research organizations: perspectives on interdisciplinarity, nurturing leadership, and integrated structure and cultures In: Weingart P, Stehr N, editors. Practising Interdisciplinarity. Toronto: University of Toronto Press; 2000 pp. 215–244.

[10] Gunn J. A few good men: the Rockefeller approach to population, 1911–1936 In: Richardson T, Fisher D, editors. The development of the social sciences in the United States and Canada: the role of Philantrophy. Ablex Publishing Corporation; 1999 pp. 97–114.

[11] Bruhn JG. Beyond discipline: creating a culture for interdisciplinary research. Integrative Physiological and Behavioural Science. 1995;30: 331–341. 10.1007/BF02691605 8788229

[12] Page SE. The difference. How the power of diversity creates better groups, firms, schools, and societies. Princenton, NJ: Princeton University Press; 2007.

[13] Huutoniemi K, Klein JT, Bruun H, Hukkinen J. Analyzing interdisciplinarity: Typology and indicators. Research Policy. 2010;39: 79–88.

[14] Nightingale P, Scott A. Peer review and the relevance gap: ten suggestions for policy makers. Science and Public Policy. 2007;34: 543–553.

[15] Molas-Gallart J, Tang P, Rafols I. The relationship between interdisciplinarity and societal impact. Journal of Science Policy and Research Management. 2014;29: 69–89.

[16] Waltman L, van Eck NJ, Van Leeuwen TN, Visser MS, van Raan AFJ. Towards a new crown indicator: an empirical analysis. Scientometrics. 2011;7: 467–481. 10.1007/s11192-011-0354-5 PMC3081055 21654898

[17] Morillo F, Bordons M, Gómez I. Interdisciplinarity in science: A tentative typology of disciplines and research areas. Journal of the American Society for Information Science and Technology. 2003;54: 1237–1249.

[18] Wagner CS, Roessner JD, Bobb K, Klein JT, Boyack KW, Keyton J, et al Approaches to understanding and measuring interdisciplinary scientific research (IDR): A review of the literature. Journal of Informetrics. 2011;5: 14–26.

[19] Porter AL, Rafols I. Is science becoming more interdisciplinary? Measuring and mapping six research fields over time. Scientometrics. 2009;81: 719–745.

[20] Rafols I, Meyer M. Diversity and network coherence as indicators of interdisciplinarity: Case studies in bionanoscience. Scientometrics. 2010;82: 263–287.

[21] Stirling A. A general framework for analysing diversity in science, technology and society. Journal of The Royal Society Interface. 2007;4: 707–719. 10.1098/rsif.2007.0213 PMC2373389 17327202

[22] Turner S. What are disciplines? And how in interdisciplinary different? In: Weingart P, Stehr N, editors. Practising Interdisciplinarity. Toronto: University of Toronto Press; 2000 pp. 46–65.

[23] Molas-Gallart J, Salter A. Diversity and excellence: considerations on research policy. IPTS Report. 2002;66.

[24] Rafols I, Leydesdorff L, O’Hare A, Nightingale P, Stirling A. How journal rankings can suppress interdisciplinarity. The case of innovation studies and business and management. Research Policy. 2012;41: 1262–1282.

[25] Stirling A. On the economics and analysis of diversity. SPRU Electronic Working Papers. 1998;28. Available: http://www.sussex.ac.uk/Units/spru/publications/imprint/sewps/sewp28/sewp28.pdf.

[26] Lowe P, Phillipson J. Reflexive interdisciplinary research: the making of a research programme on the rural economy and land use. Journal of Agricultural Economics. 2006;57: 165–184.

[27] Gibbons M. Science’s new social contract with society. Nature. 1999;402: c81–c84. 10591229 10.1038/35011576

[28] Abdool Karim SS. Stigma impedes AIDS prevention. Nature. 2011;474: 29–31. 10.1038/474029a 21637237

[29] Van Rijnsoever FJ, Hessels LK. Factors associated with disciplinary and interdisciplinary research collaboration. Research Policy. 2011;40: 463–472.

[30] Carayol N, Thi TUN. Why do academic scientists engage in interdisciplinary research? Research Evaluation. 2005;14: 70–79.

[31] Barry A, Born G, Weszkalnys G. Logics of interdisciplinarity. Economy and Society. 2008;37: 20–49.

[32] Corsi M, Ippoliti CD’, Lucidi F. Pluralism at Risk? Heterodox Economic Approaches and the Evaluation of Economic Research in Italy. American Journal of Economics and Sociology. 2010;69: 1495–1529. 10.1111/j.1536-7150.2010.00754.x

[33] Phillips N. The slow death of pluralism. Review of International Political Economy. 2009;16: 85–94.

[34] Willmott H. Listing perilously. Organization. 2011;18: 447–448.

[35] Katz JS, Martin BR. What is research collaboration? Research Policy. 1997;26: 1–18.

[36] Cummings JN, Kiesler S. Collaborative research across disciplinary and organizational boundaries. Social Studies of Science. 2005;35: 733–722.

[37] Rafols I. Strategies for knowledge acquisition in bionanotechnology—Why are interdisciplinary practices less widespread than expected? Innovation-the European Journal of Social Science Research. 2007;20: 395–412.

[38] Abbot A. Chaos of disciplines. Chicago: The University of Chicago Press; 2001.

[39] Bruce A, Lyall C, Tait J, Williams R. Interdisciplinary integration in Europe: the case of the Fifth Framework programme. Futures. 2004;36: 457–470.

[40] Laudel G, Origgi G. Introduction to a special issue on the assessment of interdisciplinary research. Research Evaluation. 2006;15: 2–4.

[41] Klein JT. Evaluation of interdisciplinary and transdisciplinary research: a literature review. American journal of preventive medicine. 2008;35: S116–S123. 10.1016/j.amepre.2008.05.010 18619391

[42] Mallard G, Lamont M, Guetzkow J. Fairness as Appropriateness : Negotiating Epistemological Differences in Peer Review. Science, Technology and Human Values. 2009;34: 573–606.

[43] Llerena P, Meyer-Krahmer F. Interdisciplinary research and the organization of the university: general challenges and a case study In: Geuna A, Salter AJ, Steinmueller WE, editors. Science and Innovation Rethinking the Rationales for Funding and Governance. Cheltenham: Edward Elgar; 2004 pp. 69–88.

[44] van Eck NJ, Waltman L, van Raan AFJ, Klautz RJM, Peul WC. Citation Analysis May Severely Underestimate the Impact of Clinical Research as Compared to Basic Research. PLoS ONE. 2013;8: e62395 10.1371/journal.pone.0062395 23638064 PMC3634776

[45] Ioannidis JPA, Boyack KW, Small H, Sorensen AA, Klavans R. Bibliometrics: Is your most cited work your best? Nature. 2014;514: 561–562. 10.1038/514561a 25355346

[46] Rinia EJ, Van Leeuwen T, Van Buren HG, Van Raan AFJ. Influence of interdisciplinarity on peer-review and bibliometric evaluations in physics research. Research Policy. 2001;30: 357–361.

[47] Levitt J, Thelwall M. Is multidisciplinary research more highly cited? A macrolevel study. Journal of the American Society for Information Science and Technology. 2008;59: 1973–1984.

[48] Larivière V, Gingras Y. On the relationship between interdisciplinarity and scientific impact. Journal of the American Society for Information Science and Technology. 2010;61: 126–131.

[49] Steele TW, Stier JC. The Impact of Interdisciplinary Research in the Environmental Sciences: A Forestry Case Study. Journal of the American Society for Information Science. 2000;51: 476–484.

[50] Adams J, Jackson L, Marshall S. Bibliometric analysis of interdisciplinary research. Report to the Higher Education Funding Council for England. Leeds: Evidence; 2007.

[51] Uzzi B, Mukherjee S, Stringer M, Jones B. Atypical combinations and citation impact. Science. 2013;342: 468–472. 10.1126/science.1240474 24159044

[52] Lariviere V, Haustein S, Boerner K. Long-Distance Interdisciplinarity Leads to Higher Scientific Impact. Plos One. 2015;10: e0122565 10.1371/journal.pone.0122565 25822658 PMC4379013

[53] Boyack KW, Klavans R. Boyack, K. W., Klavans, R. (2014). Atypical combinations are confounded by disciplinary effects. STI 2014 Leiden Conference, 64–70. Available http://sti2014.cwts.nl/download/f-y2w2.pdf. STI Indicators Conference Proceedings. 2014; 64–70.

[54] Leydesdorff L, Chen C, Rafols I. Interactive Overlays of Journals and the Measurement of Interdisciplinarity on the basis of Aggregated Journal-Journal Citations. Journal of the American Society for Information Science and Technology. 2013;64: 2573–2586.

[55] Chavarro D, Tang P, Rafols I. Interdisciplinarity and local issue research: evidence from a developing country. Research Evaluation. 2014;23: 195–209. 10.1093/reseval/rvu012

[56] Waltman L, van Eck NJ. A systematic empirical comparison of different approaches for normalizing citation impact indicators. Journal of Informetrics. 2013;87: 833–849.

[57] Zhang L, Rousseau R, Glänzel W. Diversity of references as an indicator of the interdisciplinarity of journals: Taking similarity between subject fields into account. Journal of the Association for Information Science and Technology. 2015; 10.1002/asi.23487

[58] Narin F, Stevens K, Whitlow ES. Scientific co-operation in Europe and the citation of multinationally authored papers. Scientometrics. 1991;21: 313–323.

[59] Peters HP, Van Raan AFJ. On determinants of citation scores: a case study in chemical engineering. Journal of the American Society for Information Science and Technology. 1994;45: 39–49.

[60] Katz S, Hicks D. How much is a collaboration worth? A calibrated bibliometric model. Scientometrics. 1997;40: 541–554.

[61] Wang J, Thijs B, Glänzel W. Interdisciplinarity and Impact: Distinct Effects of Variety, Balance and Disparity. Plos One. 2015;10: e0127298 10.1371/journal.pone.0127298 26001108 PMC4441438

[62] Frenken K, Hardeman S, Hoekman J. Spatial scientometrics: Towards a cumulative research program. Journal of Informetrics. 2009;3: 222–232.

[63] Fleming L. Recombinant uncertainty in technological search. Management Science. 2001;47: 117–132. 10.1287/mnsc.47.1.117.10671

[64] Nooteboom B, Van Haverbeke W, Duysters G, Gilsing V, van den Oord A. Optimal cognitive distance and absorptive capacity. Research Policy. 2007;36: 1016–1034. 10.1016/j.respol.2007.04.003

[65] Sarewitz D. How science makes environmental controversies worse. Environmental Science & Policy. 2004;7: 385–403.

[66] Hessels LK, van Lente H, Grin J, Smits REHM. Changing struggles for relevance in eight fields of natural science. Industry and Higher Education. 2011;25: 347–357.

[67] Hessels LK, Grin J, Smits REHM. The effects of a changing institutional environment on academic research practices: three cases from agricultural science. Science and Public Policy. 2011;38: 555–568.

[68] Sarewitz D, Pielke RA. The neglected heart of science policy: reconciling supply of and demand for science. Environmental Science & Policy. 2007;10: 5–16.

