# Supplementary material for: Does Interdisciplinary Research Lead to Higher Citation Impact? The Different Effect of Proximal and Distal Interdisciplinarity
Source: PLoS One. 2015 Aug 12;10(8):e0135095. doi: 10.1371/journal.pone.0135095 (PMC4534379; doi:10.1371/journal.pone.0135095)
Supplement: S1 File — (DOCX) [file pone.0135095.s001.docx]

Table A: Tobit estimates for the effect of variety, balance and disparity, controlling for number of references: *N_refer_small* and *N_refer_large.*

|  | **Dependent Variable: ln (NCS)** | | | |
| --- | --- | --- | --- | --- |
| **Variables** | (1) | (2) | (3) | (4) |
| Variety | 0.083 *** | 0.543 *** | 0.072 *** | 0.084 *** |
|  | (0.021) | (0.053) | (0.021) | (0.021) |
| Balance | -0.088 *** | -0.125 *** | 0.351 *** | -0.083 *** |
|  | (0.016) | (0.017) | (0.054) | (0.018) |
| Disparity | -0.047 *** | -0.068 *** | -0.005 | -0.092 |
|  | (0.016) | (0.017) | (0.018) | (0.071) |
| Variety^2^ | --- | -0.699 *** | --- | --- |
|  |  | (0.074) |  |  |
| Balance^2^ | --- | --- | -0.363 *** | --- |
|  |  |  | (0.042) |  |
| Disparity^2^ | --- | --- | --- | 0.041 |
|  |  |  |  | (0.064) |
| N_refer_small | -0.203 *** | -0.191 *** | -0.192 *** | -0.203 *** |
|  | (0.005) | (0.005) | (0.005) | (0.005) |
| N_refer_large | 0.152 *** | 0.151 *** | 0.145 *** | 0.152 *** |
|  | (0.005) | (0.005) | (0.006) | (0.005) |
| N_authors | 0.017 *** | 0.017 *** | 0.017 *** | 0.017 *** |
|  | (0.001) | (0.001) | (0.001) | (0.001) |
| N_Institutions | 0.008 *** | 0.009 *** | 0.009 *** | 0.008 *** |
|  | (0.003) | (0.003) | (0.003) | (0.003) |
| Internat_collab | 0.010 | 0.009 | 0.009 | 0.010 |
|  | (0.007) | (0.007) | (0.007) | (0.007) |
| National_collab | 0.008 | 0.007 | 0.008 | 0.008 |
|  | (0.006) | (0.006) | (0.006) | (0.006) |
| CBiol | -0.120 *** | -0.124 *** | -0.113 *** | -0.120 *** |
|  | (0.007) | (0.007) | (0.007) | (0.007) |
| EEE | 0.127 *** | 0.138 *** | 0.133 *** | 0.127 *** |
|  | (0.006) | (0.006) | (0.006) | (0.006) |
| FST | 0.008 | 0.009 | 0.009 | 0.008 |
|  | (0.007) | (0.007) | (0.007) | (0.007) |
| Constant | 0.462 *** | 0.445 *** | 0.324 *** | 0.469 *** |
|  | (0.019) | (0.019) | (0.025) | (0.022) |
| N. obs. | 62408 | 62408 | 62408 | 62408 |
| Log-Likelihood | -45697.0 | -45652.7 | -45660.4 | -45696.8 |
| LR χ^2^ | 6137.2 *** *** | 6225.8 *** | 6210.5 *** | 6137.7 *** |

Notes: * *p* < 0.1; ** *p* < 0.05; *** *p* < 0.01. Standard errors are in parenthesis. Eight dummies have been included in the regression to account for the effect of countries (from the authors’ affiliations) in the number of citations received. These dummies are not reported in the Table.
